# Bovine congenital erythropoietic protoporphyria in a crossbred limousin heifer in Ireland

**DOI:** 10.1186/s13620-015-0044-3

**Published:** 2015-07-02

**Authors:** Conor G. McAloon, Michael L. Doherty, Henry O’Neill, Michael Badminton, Eoin G. Ryan

**Affiliations:** School of Veterinary Medicine, University College Dublin, Dublin, Ireland; Donnington Grove Veterinary Surgery, Newbury, Berkshire, UK; Cardiff Porphyria Service, Cardiff and Vale University Health Board, Wales, UK

**Keywords:** Protoporphyria, Photosensitisation, Seizures, Convulsions, Cattle

## Abstract

An unusual case of an 11-month-old, black Limousin-cross heifer, with an 8-month history of episodic seizures and photosensitisation, was referred by a veterinary practitioner to the Farm Animal Section of the UCD Veterinary Hospital, School of Veterinary Medicine, University College Dublin, Ireland, in August 2014. Following an investigation, a diagnosis of Bovine Congenital Erythropoietic Protoporphyria (BCEPP) was made. To the authors’ knowledge this is the first report of such a case in Ireland. BCEPP should be considered as a differential diagnosis in young animals displaying periodic seizures and/or photosensitisation.

**Electronic supplementary material:**

The online version of this article (doi:10.1186/s13620-015-0044-3) contains supplementary material, which is available to authorized users.

## Background

Bovine Congenital Erythropoietic Protoporphyria (BCEPP) is an inherited condition which is most commonly reported in Limousin cattle [1], but has also been described in the Blonde d’Aquitane breed [2]. BCEPP is caused by a deficiency in the activity of ferrochelatase. This enzyme is involved in the final stage of the 8-step haem biosynthesis pathway, catalysing the chelation of ferrous iron to protoporphyrin in the production of haem [3].

Excess protoporphyrin is lipophilic and accumulates in cellular membranes [4]. The molecule absorbs light in a range of wavelengths and energy absorbed from this light can be transferred to oxygen resulting in a reactive oxygen species that may interact with proteins, lipids or DNA [4, 5].

Erythropoietic Protoporphyria was first reported in humans in 1961 [6] and in cattle in the US in 1977 [7]. The disease has since been reported in the UK [8], Australia [9], France [1] and New Zealand [10].

BCEPP is thought to be inherited in an autosomal recessive pattern [3]. Photosensitisation is the prevailing clinical presentation and results of the formation of reactive oxygen species [7]. However, more recently, case reports have been published in which the initial presenting complaint was primarily neurological [2, 11, 12]. This phenomenon appears to be associated with the bovine and not the human form of the disease [13].

This case report discusses the investigation of a case of Bovine Congenital Erythropoietic Protoporphyria (CBEPP) in a crossbred Limousin heifer, a condition that has not yet been reported in Ireland.

## Case presentation

An 11-month-old black Limousin-cross heifer from a 20-cow commercial suckler farm in County Tyrone presented with an 8-month history of episodic seizures and a 3-month history of skin lesions. Neurological signs were first observed when the animal was 3 months of age with repeat episodes occurring every 1–2 months. The farmer reported seeing the calf in lateral recumbency with paddling movements of all four limbs. The onset of this behaviour was not observed, but a period of altered mentation and ataxia was noted after the event, which the farmer recorded on video (Additional files 1 and 2). Treatment with intravenous dexamethasone (Colvasone, Norbrook, 0.08 mg/kg) and intramuscular multivitamins (Multivitamin Solution for Injection, Norbrook, 10mls) initially appeared successful but repeated events occurred.

The development of skin lesions on the rump of the calf was noticed by the farmer at approximately 8 months of age, soon after the animal went to pasture. A diagnosis of photosensitisation was made by the referring veterinarian. After a number of repeat seizures, the case was referred to the Farm Animal Section of the UCD Veterinary Hospital for further investigation.

**Fig. 1:**
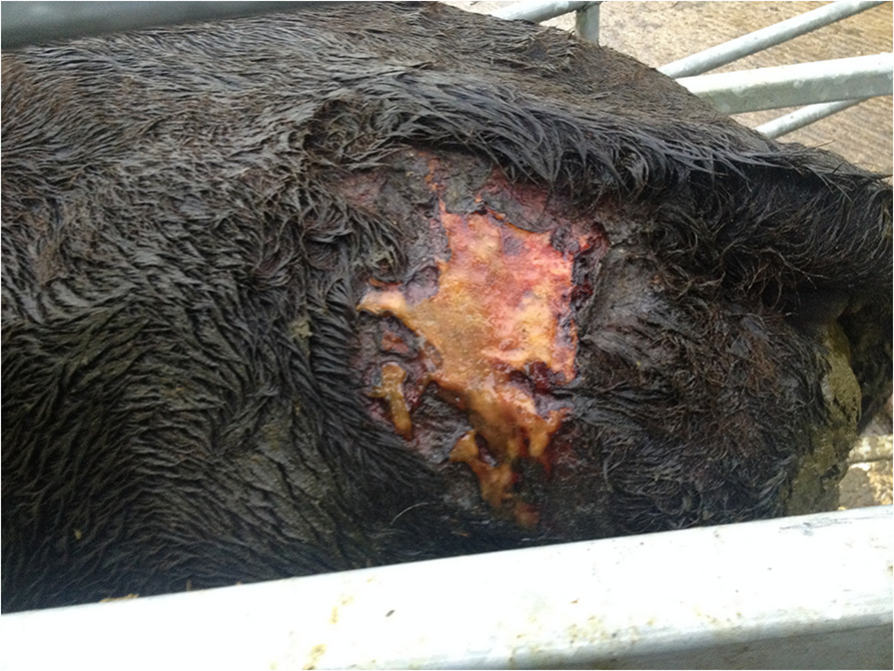
Erosion and moist exudative dermatitis in the lumbosacral area of a calf with bovine congenital erythropoietic protoporphyria

**Fig. 2:**
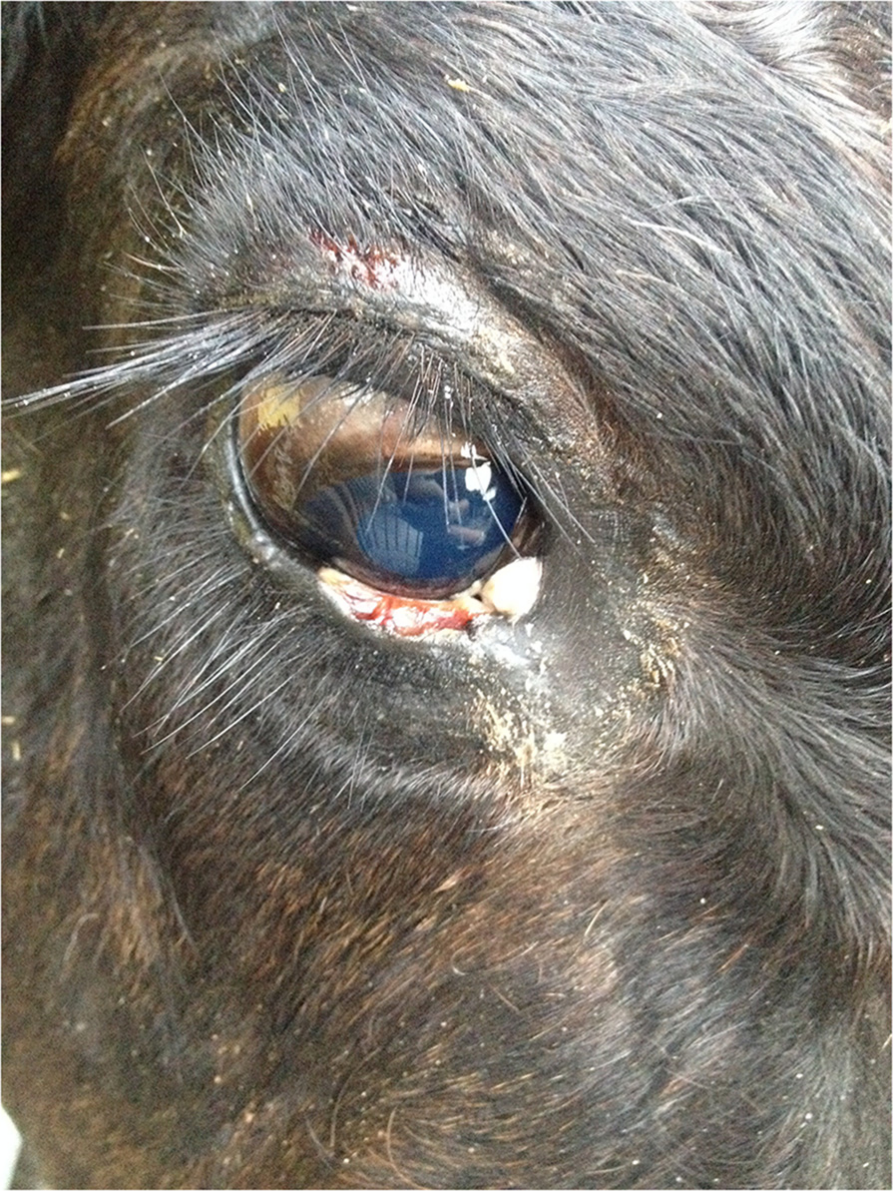
Ulceration on the palpebral margin of a calf with bovine congenital erthropoietic protoporphyria

On clinical examination, the animal had a body condition score of 2.75 (out of 5). There was a large, irregularly shaped area of erosion with moist exudation and hair loss measuring approximately 15 cm x 10 cm in the lumbosacral area, to the left of the midline (Fig. 1). A second area of crusting and alopecia was found on the right hindlimb, just caudal to the stifle on the lateral aspect of the thigh. There was further crusting and alopecia on the points of the elbows. There was ulceration of the medial aspect of the right lower palpebra and crusting of the upper palpebra (Fig. 2), thickening and loss of pigment of the medial aspect of the lower lid of the left eye and tear staining indicative of epiphora bilaterally.

The animal was bright and alert on presentation. Neurological examination was unremarkable apart from facial and nasal hypalgesia bilaterally. Heart rate, respiratory rate and temperature were within normal limits. The heifer was notably smaller than pen-mates. Two videos were presented by the farmer, both of which were taken subsequent to separate seizure events (Additional files 1 and 2). In the first video the calf demonstrates a crouched posture with stiffness and hypermetria of the forelimbs. In the second video the calf exhibits ataxia and hypermetria of the forelimbs.

At this stage, photosensitisation (Type 1, 2 or 3) was considered the most likely cause of the dermatological abnormalities, although uncommon immune-mediated dermatoses were also regarded as possible. Differential diagnoses for the neurological signs were less defined at this stage but included cerebrocortical necrosis (CCN), brain abscessation, meningitis, lead poisoning, epilepsy and neurological signs associated with some causes of type-2 photosensitisation.

Haematological parameters were within normal limits. Biochemistry revealed slight elevations in Gamma-Glutamyl Transferase (GGT) (80 U/L, reference range 0–36 U/L), and Glutamate Dehydrogenase (GLDH) (91 U/L, reference range 0–19 U/L) as well as a small decrease in serum albumin levels (26.3 g/L, reference range 29–39 g/L).

A faecal sample was obtained and examined for the presence of *Fasciola hepatica,* no eggs were observed in this instance.

In order to assess levels of protoporphyria, a blood sample was obtained in an EDTA container and sent to the Cardiff Porphyria Service, Cardiff and Vale University Health Board, Wales, UK. The sample was tested using fluorescence emission spectroscopy and showed a prominent porphyrin peak with an emission maximum at 627 nm consistent with a diagnosis of Bovine Congenital Erythropoietic Protoporphyria.

## Discussion

Serum biochemistry revealed only modest changes. GGT in cattle is increased principally due to cholestatic disease but also in the case of hepatocellular disease with cholestasis as a secondary feature [14]. GGT is commonly increased in animals with fasciolosis [15]. Although no eggs were discovered on faecal examination, fasciolosis could not be ruled out as a cause of GGT elevation given the poor sensitivity of this diagnostic method [16]. Glutamate Dehydrogenase (GLDH) is typically increased as a result of hepatocyte damage [17].

Hypoalbuminaemia occurs as a result of loss of albumin or insufficient albumin synthesis—often as a result of inflammation or hepatic insufficiency [17]. However, liver disease must be chronic and severe in order to result in hypoalbuminaemia [18]. In addition, cattle with chronic debilitating disease may be hypoalbuminaemic with low or normal total protein [19].

The serum biochemistry profile suggested possible liver involvement in this case. Interestingly in human patients, mild elevations in liver enzymes are reported to occur in around 20 % of cases of protoporphyria with hepatic failure occurring in less than 5 % [20]. Mild elevations in GLDH were also reported in a previous bovine case study in the UK [11].

Photosensitisation is a relatively common presentation in cattle in Ireland. The condition is caused by the build-up of photodynamic agents in the skin resulting in dermatitis when exposed to light [21]. Three forms of photosensitisation are recognised; Type 1-primary photosensitisation, Type 2-photosensitisation due to aberrant pigment synthesis and Type 3-hepatogenous photosensitisation [22].

Primary photosensitisation occurs as a result of the ingestion of exogenous photodynamic agents including hypericin in *Hypericum perforatum* (St. John’s Wort), fagopyrin in *Fagopyrum esulentum* (Buckwheat), perloline in *Lolium perenne* (perennial ryegrass) and *Secale cereal* (annual ryegrass) and furanocoumarins [21, 22].

Type-2 photosensitisation occurs in ‘the porphyrias’, a group of disorders characterised by defective haem synthesis. Congenital erythropoietic porphyria (BCEP) and congenital erythropoietic protoporphyria (BCEPP) have both been reported in domestic cattle [11, 23]. Accumulation of uroporphyrin I and coproporphyrin I results in type-2 photosensitisation in BCEP [21, 23, 24], whereas protoporphyrin is the cause of photosensitisation in BCEPP [22].

Type-3 (hepatogenous) photosensitisation is the most common form of photosensitisation [22, 25]. In this case, a normal end product of chlorophyll metabolism, phylloerythrin, is the photodynamic agent. Phylloerythrin is normally absorbed from the gastrointestinal tract and excreted in bile, however, accumulation of the substance may result from obstruction of biliary secretion caused by hepatitis or bile duct obstruction [21]. Chronic fasciolosis is commonly linked to Type-3 photosensitisation in Ireland.

Pyrrolizidine alkaloid toxicosis caused by *Senecio jacob*ea (Ragwort) causes extensive hepatocellular damage and type-3 photosensitisation occurs in a proportion of these cases [22]. Sporidesmin produced by *Pithomyces chartarum* is associated with damage to biliary epithelium in cattle, resulting in type-3 photosensitisation. The resulting condition, known as ‘facial eczema’ in New Zealand, Australia and South Africa, is rare outside these countries despite the cosmopolitan nature of the fungus [22].

Photosensitisation of unknown aetiology has been associated with consumption or rape, kale, mouldy alfalfa hay or silage, grazing lush pasture and following induction of parturition by corticosteroid use [21].

Seizures are defined as the physical expression of abnormal electrical discharges in forebrain neurons, causing spontaneous, paroxysmal involuntary movements and are a rare presentation in cattle since this species is known to have a relatively high seizure threshold [26]. Differential diagnoses include; hypomagnesaemia [27], acute hypernatraemia [28], nervous signs associated with coccidiosis [29], some presentations of lead poisoning, Aujesky’s disease, brain abscessation, suppurative meningoencephalitis or cerebrocortical necrosis (CCN), [30, 31], hypovitaminosis A [32], inherited idiopathic epilepsy of cattle [21, 33], and bovine familial convulsions and ataxia [34]. Congenital pseudomyotonia [35], whilst not associated with seizures or photosensitisation, may result in a rigid and uncoordinated gate similar to those captured on video by the famer (Additional files 1 and 2).

A combination of neurological signs and photosensitisation is observed in animals with hepatoencephalopathy. Rare congenital conditions such as portosystemic shunt may cause hepatic encephalopathy in calves [36] with many animals displaying signs such as dullness, ataxia, tenesmus, aimless wandering etc. [37]. However seizures are not a common manifestation of this disease in cattle. Similarly, chronic ragwort poisoning leads to liver failure, hepatic encephalopathy and photosensitisation, but seizures are not commonly associated with this disease.

Animals with Bovine Erythropoietic Protoporphyria may present with photosensitisation and neurological signs including seizures [12]. Seizures are not associated with the human form of the disease [13] and the cause of the neurological signs in cattle is unclear. BCEPP is thought to be inherited in an autosomal recessive manner in cattle [3], whereas in humans, Erythropoietic Protoporphyria is more commonly inherited as a dominant disorder with low clinical penetrance [38]. A more rare autosomal recessive form has been reported in humans and affected individuals are reported to demonstrate unusual dermatological changes with a subgroup displaying neurological symptoms [39].

Neurological disturbances in humans are reported with acute forms of porphyria. Initially these symptoms were attributed to accumulation of 5-aminolevulinic acid. This molecule inhibits γ-amino-butyric acid (GABA) a primary inhibitory neurotransmitter of the central nervous system [40]. However, it is now believed that although this molecule may be responsible for neuropathic pain observed in affected individuals [41], seizures are more likely attributed to metabolic imbalances such as hyponatraemia [42].

**Fig. 3:**
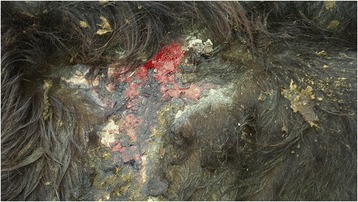
Area of ulceration in the lumbosacral area of a calf with bovine congenital erythropoietic protoporphyria, 6 months after housing

A diagnosis of BCEPP was reached in this case following fluorescent spectroscopy of plasma. Subsequently the herdowner was advised to keep the animal indoors permanently. A follow-up examination was undertaken 6 months later and although the animal had gained weight at this stage, it was still considerably smaller than cohort animals. Skin lesions had improved, however one particular area remained ulcerated (Fig. 3) and hair had not regrown on many of the affected areas. No further seizures were observed.

Females in this herd were bred by natural service to a stock bull. However, the sire of this heifer was not intended as a breeding animal and had been slaughtered prior to presentation of the case. Protoporphyria is considered an autosomal recessive condition. Given that this case was the only calf in the herd sired by this particular bull, and the assumed rarity of this condition in Ireland, further cases in the herd were considered unlikely. The herdowner was advised not to breed from the affected animal. As an additional precaution, removal of the dam from the herd was advised.

## Conclusions

Periodic seizures is an uncommon presentation in young bovine animals. However, photosensitisation is a common presentation in cattle in Ireland. It is likely that little further diagnostic work-up is undertaken in these animals. Bovine Congenital Erythropoietic Protoporphyria should be considered as a differential diagnosis in cases displaying seizures and/or photosensitisation, particularly in the Limousin breed.

## Additional files

Additional file 1:
**Crouched posture and forelimb hypermetria following a seizure in a calf with bovine congenital erythropoietic protoporphyria.**
Additional file 2:
**Hindlimb ataxia and forelimb hypermetria in a calf with bovine congenital erythropoietic protoporphyria.**
